# Endometrial osseous metaplasia presented as infertility cases with intra-operative obstacles: Two case reports and literature review

**DOI:** 10.5339/qmj.2025.24

**Published:** 2025-01-21

**Authors:** Zeena Helmi, Wassan Nori

**Affiliations:** ^1^Department of Obstetrics and Gynecology, Mustansiriyah University, Baghdad, Iraq *Correspondence: Wassan Nori. Email: dr.wassan76@uomustansiriyah.edu.iq

**Keywords:** Endometrial osseous metaplasia, abortion, curettage, infertility, hysteroscopy, diagnostic procedures

## Abstract

**Background:**

Endometrial osseous metaplasia (OM) is a rare condition characterized by the transformation of endometrial tissue into bone cells. Despite its rarity, OM remains a significant contributor to infertility. Although the underlying mechanism remains debatable, an association with previous abortions and curettage has been suggested.

**Case presentation:**

We present two cases of OM presented to the infertility clinic and discuss their similarities and discrepancies in presentation and risk factors. A transvaginal ultrasound raises suspicion about the diagnosis of OM with a hyperechoic mass and post-acoustic shadowing. An office hysteroscopy showed white, mesh-like bony sheets. Both cases underwent operative hysteroscopy to address surgical challenges, and the two cases were followed postoperatively for one year.

**Discussion:**

A comprehensive literature review examined various aspects of OM, including diagnosis, therapeutic options, outcomes, prognosis, and follow-up. Our aim was to raise awareness of this intriguing condition by providing up-to-date knowledge and emphasizing the central role of hysteroscopy in diagnosis and treatment. Here, we present two cases with the same complaint, infertility. Moreover, although the same treatment method was used in both cases, only one achieved pregnancy. This highlights that OM is a possible underlying cause of infertility, in addition to considering other factors that contribute to the overall clinical picture.

**Conclusion:**

OM should be considered in the evaluation of infertility despite its rarity, especially with hyperechoic lesions and acoustic shadowing on ultrasound examination. Hysteroscopy is the gold standard for diagnosis and therapeutic approaches. A complete understanding of the reasons that trigger its growth is crucial. To rule out other differential diagnosis, a holistic evaluation of the patient's history, imaging, and histopathological examination is needed.

## Introduction

Metaplasia is the transformation of one cell type into another within the same tissue. It is reported in various parts of the body, including the endometrium. Endometrial osseous metaplasia (OM) occurs when bone cells are found in the endometrial lining of the uterus. It is a very rare condition with an incidence of three cases per 10,000 females.^1^

It is still unclear what triggers the transformation of the endometrium into bony cells, and many researchers have proposed a hypothesis to shed light on its occurrence. Most acceptable ones revolve around OM associated with a history of miscarriage and previous dilatation and curettage (D&C) procedures,^2^ which might trigger a local inflammatory reaction leading to cell transformation. Others proposed a chronic inflammation such as tuberculosis or pyometra as an underlying mechanism in which cell transformation may be an adaptive mechanism to the change in the uterine environment. Pathologists proposed that chronic stimulation of the endometrium by estrogen and metabolic disease is a triggering factor for endometrial metaplasia.^1,2^

In some affected women, it is asymptomatic and discovered accidentally; others tend to present with infertility as the chief complaint.^3^ Due to its rarity, OM represents a diagnostic challenge. Imaging and hysteroscopy play key roles in the diagnosis, which is confirmed by histological examination.^4^

Here, we present two cases of endometrial OM that occurred in the infertility clinic. We discuss their clinical presentation, diagnostic challenges, and therapeutic obstacles faced during hysteroscopic treatment. Additionally, we comprehensively review the literature and address the presentation, investigation, therapeutic options, prognosis, and follow-up of endometrial OM. In fact, there is a gap in current knowledge of diagnostic guidelines for OM and an inconsistency in the surgical techniques used. Moreover, it is unclear what impact OM removal has on endometrial regeneration and, if any, scarring, especially with respect to fertility odds following its removal. Our aim was to provide up-to-date knowledge to raise awareness of this underestimated cause of infertility and emphasize the invaluable role of hysteroscopy in its management.

## Case Presentation

### First case

A 44-year-old Iraqi woman, G3P2A1, attended the outpatient clinic of Al Yarmouk Teaching Hospital in Baghdad, Iraq, for 7 years seeking advice for secondary infertility. She had a regular menstrual cycle and never used contraceptives. Her only pregnancy was 8 years ago, which ended in a first-trimester miscarriage and required D&C. The investigation carried out at that time could not reveal the cause of the abortion. History of sexually transmitted infections (STIs) was negative.

A pelvic examination showed a normal anteverted uterus and adnexa. Whitish leukorrhea was noted without cervical tenderness. Investigations were within normal range, including blood count, biochemistry, and urinalysis. On day 2 of the menstrual cycle, the hormonal profile showed normal FSH, LH, prolactin, and testosterone levels (6.2 IU/L, 4.7 IU/L,17.39 ng/ml, and 0.63 nmol/L, respectively). Furthermore, her partner's seminal fluid analysis was normal. She had a hysterosalpingography (HSG) performed 5 years ago, showing normal uterine cavity and patent fallopian tubes. She refused the advice to repeat it. She received no induction of ovulation during the period of secondary infertility.

**Figure 1.**
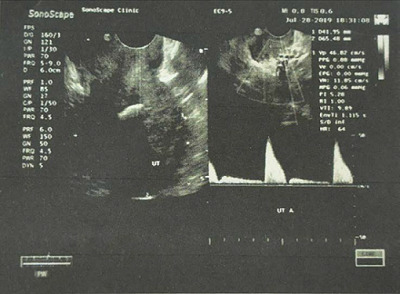
Transvaginal ultrasound showing an intrauterine hyperechogenicity with post-acoustic shadowing suggestive of calcification associated with chronic endometritis.

**Figure 2.**
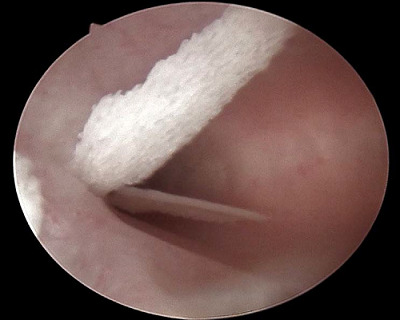
Diagnostic office hysteroscopy showing an adequate endometrial cavity with multiple white, mesh-like boney sheets of varying lengths (5–25 mm) occupying the uterine cavity.

A transvaginal ultrasound (TVUS) showed intrauterine hyperechogenicity, suggesting chronic endometritis with associated calcification (Figure 1). The patient's serum calcium, phosphorus, and parathyroid hormone levels were checked and found to be normal. To confirm the case, a diagnostic office hysteroscopy was performed using a scope of 2.9 mm diameter from Karl Storz Germany, a 30-degree oblique lens, an outer sheath of 5 mm diameter, a xenon light source, a constant-flow hysteroscopy pump, a distension medium of normal saline 0.9%, and pressure 80–100 mmHg without anesthesia or pre-procedure medication. Hysteroscopy revealed an adequate endometrial cavity with multiple white, mesh-like bony sheets of varying lengths (5–20 mm) filling the entire uterine cavity (Figure 2).

**Figure 3.**
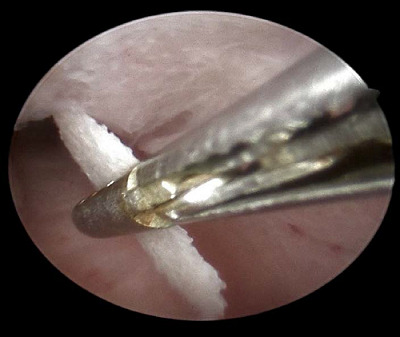
Endometrial osseous metaplasia lesions completely removed with hysteroscopic forceps.

The case was admitted for operative hysteroscopy under GA (general anesthesia) with normal saline as distension media 2 weeks later, immediately after the menstrual cycle in the follicular phase. The endometrial OM was identified and completely removed with hysteroscopic forceps (Figure 3).

**Figure 4.**
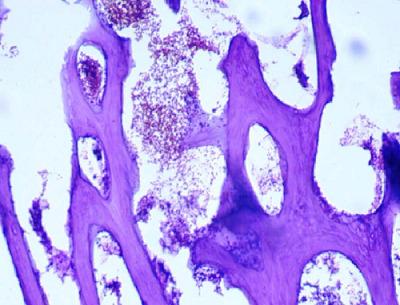
Section showing mature lamellar bone trabeculae associated with normal marrow spaces (H&E X200).

The operation went smoothly and without complications. The removed tissue was sent for histopathological examination and confirmed the diagnosis of endometrial OM with stromal edema (Figure 4).

At the follow-up visit 2 weeks later, a TVUS confirmed a normal-looking uterine cavity. Nine months after the surgery, the patient became pregnant spontaneously.

### Second case

A 37-year-old Iraqi woman presented to the outpatient clinic complaining of primary infertility that had existed for 15 years. Her gynecological history was relevant for dysmenorrhea that was unresponsive to medications such as acetaminophen or NSAIDs. She had undergone ovulation induction and her HSG was normal, which was done 4 years ago.

She had a regular menstrual cycle. Her medical and surgical history were unremarkable, and she had a negative history of STIs. The pelvic examination revealed a normal-sized, anteverted uterus and normal adnexa. There was no cervical motion tenderness. She was investigated in the same manner as case 1. None of the blood, biochemical, and hormonal tests were abnormal. Male seminal fluid analysis was normal.

**Figure 5.**
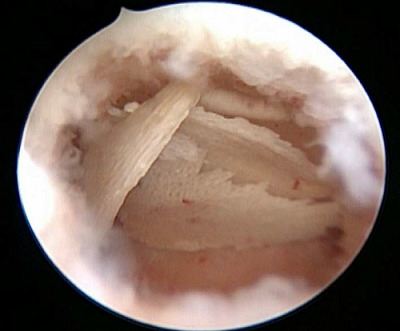
Office hysteroscopy showing a uterine cavity filled with multiple white, mesh-like boney sheets of varying lengths (5–25 mm).

TVUS showed a similar appearance to case 1: an intrauterine hyperechogenicity. To confirm the case, a diagnostic office hysteroscopy was performed using a scope of 2.9 mm diameter from Karl Storz Germany, a 30-degree oblique lens, an outer sheath of 5 mm diameter, a xenon light source, a constant-flow hysteroscopy pump, a distension medium of normal saline 0.9%, and a pressure of 80–100 mmHg without pre-procedure medication. The endometrial cavity was filled with multiple white, mesh-like bony sheets of varying lengths (5–25 mm), which filled the entire uterine cavity (Figure 5).

**Figure 6.**
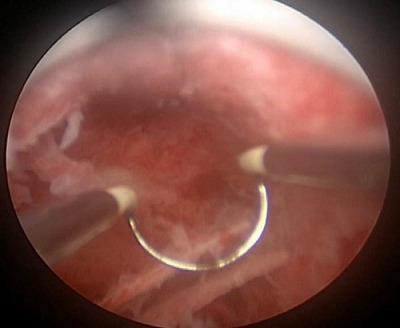
Multiple endometrial osseous lesions were completely removed using the monopolar cutting loop under hysteroscopic control.

She was scheduled for operative hysteroscopy under general anesthesia, and two weeks later with a 10 mm Storz resectoscope. After identifying the endometrial OM, an attempt to remove these bony pieces with forceps failed. In fact, two forceps were broken, and we had to remove these bony fragments using the monopolar cutting loop with hysteroscopic guidance without electrical current (Figure 6).

**Figure 7.**
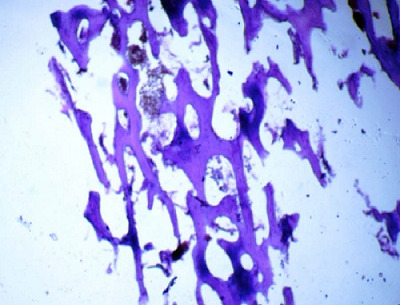
Section showing mature lamellar bone tissue with normal marrow spaces (H&E X100).

**Figure 8.**
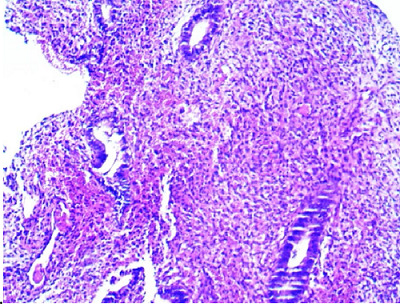
Section showing non-secretory endometrial glands associated with focal stromal edema (H&E X200).

Fortunately, there were no operative complications. Pathological examination confirmed endometrial OM with stromal edema (Figures 7 and 8). Two weeks after surgery, the patient underwent a TVUS that revealed no abnormal appearance. A second-look office hysteroscopy revealed a normal endometrium. One year after the procedure, the patient failed to conceive.

## Discussion

### Clinical presentation

Endometrial OM is described as an endogenous, non-neoplastic pathological condition. Ossification has also been reported in the ovary, vagina, cervix, and mesosalpinx, but in this study, we focus on endometrial OM.

Most often, OM is presented as infertility.^2,5^ The most common symptom of OM is infertility, which recurs after its removal in up to 70% of the cases.^2^ Other associated symptoms include menstrual abnormalities: menorrhagia or amenorrhea, sometimes irregular bleeding,^4^ dysmenorrhea, and dyspareunia.^6^ Other reported featuresinclude chronic pelvic pain, vaginal discharge, and, in some, foreign body-like sensation.^7^ Others may be asymptomatic, and the condition was discovered incidentally during an investigation.^8^

The two cases presented here suffered from infertility. The first case presented had secondary infertility, along with leukorrhea, which did not draw her attention because it occurred occasionally, was odorless, and was not associated with itching or pain. In comparison, primary infertility was present in the second case.

The reason for the infertility observed in OM cases is that it acts as an intrauterine device and prevents implantation by disrupting the endometrial surface.^6^ Additionally, their presence in the endometrium can trigger inflammatory mediators and cytokines that hinder successful implantation and conception.^7^ It is worth mentioning that most of the operated cases resulted in a successful pregnancy. However, the minority do not succeed in pregnancy, possibly due to extensive damage to the endometrium, limited regenerative power of the endometrium, and additional infertility parameters unrelated to OM.^9,10^

The pathology underlying OM is not fully understood. A comprehensive review of published cases shows that most of the cases suffer from secondary infertility,^11^ which may support the hypothesis that OM develops in response to retained fetal parts in the uterus.^1^

However, there are some cases, like case 2 reported here, who suffer from primary infertility. Some researchers discussed that ossification is simply a protective mechanism that the body develops in response to chronic inflammation, as in the study by Lee et al. reporting chlamydia and mycoplasma infections in the affected case.^12^

The chronic inflammation theory could explain the chronic pelvic pain or vaginal discharge reported in some affected women. It is worth mentioning that vaginal discharge was the predominant symptom in postmenopausal women, whereas leukorrhea was the most common symptom in women of reproductive age, and the diagnosis was confirmed by hysteroscopy.^13^

Another proposed hypothesis for cases of primary infertility is the presence of endometrial totipotent cells that can transform into other tissue types, including cartilage and bone.^14^

Conditions that stimulate such transformation may include prolonged exposure of the endometrium to estrogen stimulation as in polycystic ovaries syndrome^15^ chronic endometrial inflammation, or metabolic disorders such as hypercalcemia, hypervitaminosis D, or hyperphosphatemia.^8^

In our case, the patient was found to have mycoplasma and chlamydia infections, and a history of abortion could stimulate the formation of endometrial OM.

The study by Cayuela et al. provided supporting evidence for this theory that a DNA signature matched between the excised OM and the patient's own DNA, excluding the fetal origin of OM, which required paternal DNA.^5^

The imaging of choice for diagnosis is TVUS, which shows a hyperechoic solid lesion in the endometrium with an acoustic shadow. TVUS is superior to HSG and MRI as both may miss the case.^5,16^

Hysteroscopy is the gold standard for confirming diagnosis and treatment. Hysteroscopically, OM can be seen as white, thin calcified plaque or coral spicules, or the affected area may appear as white ivory-white ossified endometrium.^12,17^ The two cases presented had the typical TVUS mentioned above, and office hysteroscopy confirmed the diagnosis. To reach the definitive diagnosis, a triad of investigations involving ultrasound, hysteroscopy, and histopathological confirmation is required,^18^ as shown in 9.

The differential diagnosis of hyperechoic linear or irregular areas within the endometrium casting posterior acoustic shadowing on ultrasound examination includes intrauterine contraceptive devices, calcified polyp, calcified submucosal leiomyoma, mature teratoma, foreign body, endometrial tuberculosis, Asherman's syndrome, and rarities such as heterotopic bone and uterine malignant mixed Mullerian tumors.^1,2^

Treatment for OM is surgical, including D&C, hysteroscopy, or hysterectomy. Although many surgeons prefer D&C in many management contexts, others believe that it is no longer preferred and recommend hysteroscopic intervention as diagnostic and therapeutic options.^2^

When tailoring treatment for each case, it is reasonable to consider the patient's history, fertility desire, OM size, and location. In some cases, removal of the entire OM was not possible due to its larger size, multiple locations, and penetration of the entire uterine wall, and therefore hysterectomy may be considered.^19^ The main points for each method are summarized in Table 1 ^5,20,21,22^ A histopathological examination will confirm the diagnosis.

Hysteroscopic intervention (using scissors and a resectoscope) is preferred for diagnostic and therapeutic options. Typically, resection is performed using loop or diathermy, and if perforation is suspected, a second look at laparoscopy may be needed. If the remaining bony pieces are to be retrieved, the use of a laser as an energy source is proposed.^17^

Fertility retention is expected once complete resection is achieved, and the conception rate reported in the literature is 50–70%.^5,19^

In 2021, Sood et al. presented a novel treatment technique with the assistance of a urologist to benefit from their familiarity and experience with the equipment: the percutaneous nephrolithotomy. This new approach is known as Per-Cervical Utero Lithotripsy (PCUL). The latter is used to break large bony chunks into smaller pieces so that they can be easily picked up by the hysteroscope, thereby minimizing the risk of perforation.^23^

In the first case, the OM was successfully removed with forceps during operative hysteroscopy. In the second case, the intervention was ineffective, and we had to resort to an alternative. A monopolar cutting loop under hysteroscopic guidance successfully removed all bony fragments without complications. Only case 1 became pregnant after hysteroscopic removal in less than one year following the procedure, highlighting the role of office hysteroscopy in the evaluation of infertile couples.

The prognosis for OM is good after complete removal of the tissue. Symptoms are expected to resolve quickly, and fertility is expected to return in less than a year.

Recurrence is not common; however, follow-up of patients is recommended as the long-term effects of OM are not fully known due to its rarity.^1^

Early management of OM cases can alleviate its symptoms and improve patients’ chances of fertility.

## Conclusion

Although OM is a rare condition, it should be taken into account in infertility cases, especially when imaging shows hyperechoic lesions with post-acoustic shadowing. TVUS is the preferred imaging technique, and many recommend hysteroscopy as the old standard for diagnostic and therapeutic purposes. It is crucial to improve our understanding of the factors that trigger their growth – integrating history, imaging, and histopathological examination helps to rule out other possible diagnoses.

### List of abbreviations

[Table tbl2]

### Acknowledgment

We would like to thank Mustansiriyah University, Baghdad, Iraq, for its support in the present study.

### Ethical statement

We obtained approval from the Ethics Committee of Mustansiriyah University (Ref. no. MOG64; dated January 2024).

### Consent for publication and participation

Informed consent was obtained from the participants for publication of the cases and associated photographs and images in the study.

### Competing interests

The authors have no conflicts of interest to declare.

### Authors’ contribution

**ZH** and **WN** conceived and designed the study, analyzed the data, drafted the manuscript, and approved the final version of the manuscript.

## Figures and Tables

**Figure 9.**
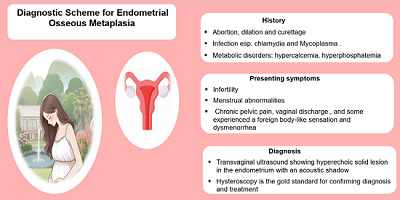
Diagnostic scheme for endometrial osseous metaplasia.

**Table 1.** Comparison of the critical point for dilatation and curettage (D&C) with hysteroscopy in OM treatment.

Parameters	D&C	Hysteroscopy

Accuracy in diagnosis	Although it is a blind procedure, the histopathological sample obtained is better than that of hysteroscopy.	It has the advantage of direct vision and unveiling hidden pathology. Definitive data on histopathological sample adequacy are lacking.

Complication and safety	Compared to hysteroscopy, there was a slightly increased risk of perforation and infection. However, it is generally safe.	It has a lower complication profile compared to D&C. Direct visualization reduces the risk of injury.

Efficiency in the removal and recurrence rate	It is not as precise as hysteroscopy. Recurrence is possible if the tissue is not completely removed.	It has better efficacy because of direct visualization. If performed well, there will be no recurrence because all the tissue is removed.

Patient outcome	Some patients may experience post-D&C problems such as tubal blockage or intrauterine adhesion.	It has the advantages of a non-invasive approach and fewer complications.

Pregnancy odds	Time-to-conception interval tends to be longer.	Time-to-conception interval tends to be shorter.


**Table tbl2:** 

D&C	Dilatation and Curettage

FSH	Follicle-Stimulating Hormone

GA	General Anesthesia

HSG	Hysterosalpingography

LH	Luteinizing Hormone

NSAIDs	Nonsteroidal Anti-Inflammatory Drugs

OM	Osseous Metaplasia

STIs	Sexually Transmitted Infections

TVUS	Transvaginal Ultrasound

